# Mean Platelet Volume (MPV) Predicts Middle Distance Running Performance

**DOI:** 10.1371/journal.pone.0112892

**Published:** 2014-11-11

**Authors:** Giuseppe Lippi, Gian Luca Salvagno, Elisa Danese, Spyros Skafidas, Cantor Tarperi, Gian Cesare Guidi, Federico Schena

**Affiliations:** 1 Laboratory of Clinical Chemistry and Hematology, Academic Hospital of Parma, Parma, Italy; 2 Laboratory of Clinical Biochemistry, Department of Life and Reproduction Sciences, University of Verona, Verona, Italy; 3 CeRiSM (Centre for Mountain Sport and Health), Rovereto (TN), Italy; 4 Department of Neurological, Neuropsychological, Morphological and Movement Sciences, University of Verona, Verona, Italy; University of the Balearic Islands, Spain

## Abstract

**Background:**

Running economy and performance in middle distance running depend on several physiological factors, which include anthropometric variables, functional characteristics, training volume and intensity. Since little information is available about hematological predictors of middle distance running time, we investigated whether some hematological parameters may be associated with middle distance running performance in a large sample of recreational runners.

**Methods:**

The study population consisted in 43 amateur runners (15 females, 28 males; median age 47 years), who successfully concluded a 21.1 km half-marathon at 75–85% of their maximal aerobic power (VO_2_max). Whole blood was collected 10 min before the run started and immediately thereafter, and hematological testing was completed within 2 hours after sample collection.

**Results:**

The values of lymphocytes and eosinophils exhibited a significant decrease compared to pre-run values, whereas those of mean corpuscular volume (MCV), platelets, mean platelet volume (MPV), white blood cells (WBCs), neutrophils and monocytes were significantly increased after the run. In univariate analysis, significant associations with running time were found for pre-run values of hematocrit, hemoglobin, mean corpuscular hemoglobin (MCH), red blood cell distribution width (RDW), MPV, reticulocyte hemoglobin concentration (RetCHR), and post-run values of MCH, RDW, MPV, monocytes and RetCHR. In multivariate analysis, in which running time was entered as dependent variable whereas age, sex, blood lactate, body mass index, VO_2_max, mean training regimen and the hematological parameters significantly associated with running performance in univariate analysis were entered as independent variables, only MPV values before and after the trial remained significantly associated with running time. After adjustment for platelet count, the MPV value before the run (p = 0.042), but not thereafter (p = 0.247), remained significantly associated with running performance.

**Conclusion:**

The significant association between baseline MPV and running time suggest that hyperactive platelets may exert some pleiotropic effects on endurance performance.

## Introduction

According to a recent on-line survey, recreational running is the most popular leisure sport activity, followed by lifting weights, biking, hiking and other outdoor activities [1]. More specifically, 75% of adults aged 24 to 44 years are engaged in outdoor running activities at least once a week in the US [2]. The typical middle distance runner is a “normal” trained adult subject, with few previous experiences in competitive sport and without special functional characteristics. The broad popularity of middle distance is mostly attributable to a variety of reasons, which include no need of special talent or highly-specialized and expensive equipment, and the remarkable benefits on health, fitness, stress reduction and weight control [2]. It is also noteworthy that the practice of habitual running has been associated with a significantly reduced risk of obesity, hypertension, diabetes, cardiovascular disease, cancer, osteoporosis, depression and several other chronic conditions, thus resulting in an overall 20% to 40% lower risk of mortality [3].

Both running economy and overall performance in middle distance running depend on a number of physiological factors, which are partially different from those required for short and long distance running [4], [5]. The published research on half-marathon runners has mainly focused on a number of specific anthropometric variables (i.e., midaxillary skinfold, body mass index, percent body fat), functional characteristics (i.e., maximal aerobic power [VO_2_max)], body core temperature), volume and intensity in training [6]–[8]. Despite the well-established relationship existing between packed cell volume, VO_2_max, aerobic performance and maximal exercise capacity [9]–[11], a fact that has also contributed to the increase use of blood doping in sports during the past decades [12], there is little information about the association between hematological variables and middle distance running performance. As such, the aim of this study was to investigate whether some hematological parameters may predict half-marathon running time in a large sample of recreational runners.

## Materials and Methods

The study was performed during a specific event called “Run For Science”, held in Verona (Italy) in April 2014, with the purpose of analyzing the normal response of adult person to middle distance running. Forty three amateur runners were recruited (15 females and 28 males; median age 47 years and IQR 42–50 years; median body mass index 23 kg/m^2^ and IQR, 22–25 kg/m^2^), who successfully concluded a 21.1 km half-marathon at 75–85% of their VO_2_max. All athletes were members of a non professional team, were habitually involved in recreational running (mean training regimen 222 min/week and IQR 191–253 min/week; maximal oxygen uptake 50 mL/kg/min and IQR 46–55 mL/kg/min), and had rested for not less than 36 hours before the trial. Maximal aerobic capacity was individually measured in the last two weeks before the event by a running test on a treadmill using a breath by breath ergospirometric system (Quark B2, Cosmed Italy). After appropriate familiarization, each runner underwent a progressive incremental test, starting from habitual running pace and increasing speed of 0.5 km/h every min till reaching the volitional exhaustion. None of the subjects were taking medications known to alter erythrocyte or platelet metabolism, including antiplatelet or antihypertensive drugs and erythropoiesis stimulating substances. The trial started at 9.30 AM and the 21.1 km distance was covered on a relatively flat route near Verona (35 m vertical gain, with maximal slope of 1.8%), in a partially sunny day with temperatures between 12–19°C and humidity between 55–75%. Participants were free to drink *ad libitum* during the run. Blood was drawn in primary blood tubes containing K_2_EDTA (Terumo Europe N.V., Leuven, Belgium) 10 min before the start of the run and immediately thereafter (i.e., within 15 min after conclusion). The whole blood samples were immediately transported to the local laboratory under controlled conditions of temperature and humidity, where a complete blood cell count (CBC) was performed on Advia 2120 (Siemens Healthcare Diagnostics, Tarrytown NY, USA), which included measurement of hematocrit, hemoglobin, red blood cell (RBC) count, mean corpuscular volume (MCV), mean corpuscular hemoglobin (MCH), mean corpuscular hemoglobin concentration (MCHC), RBC distribution width (RDW), platelet count, mean platelet volume (MPV), white blood cell (WBC) count and differential, reticulocyte count and reticulocyte hemoglobin concentration (RetCHR). The analysis of blood specimens was concluded within 2 hours after sample collection and all results were finally expressed as median and interquartile range (IQR). Differences of pre-run and post-run values were analyzed with Wilcoxon's test for paired samples. Univariate (i.e., Spearman's correlation) and multivariate analysis (with adjustment for age, sex, blood lactate, body mass index, VO_2_max, mean training regimen and CBC parameters significantly associated with running time in univariate correlation) were performed, in order to identify potential predictors of running performance. The statistical analysis was performed with Analyse-it (Analyse-it Software Ltd, Leeds, UK) for Microsoft Excel (Microsoft Corporation, Redmond, WA, USA). All subjects gave a written consent for being enrolled in this investigation. The study was approved by the local ethical committee (Department of Neurological, Neuropsychological, Morphological and Movement Sciences, University of Verona) and performed in accord with the Helsinki Declaration of 1975 (additional information can be downloaded from the institutional Website: http://www.dsnm.univr.it/?ent=iniziativa&id=5382, Last accessed, 10 October 2014).

## Results

**Figure 1:**
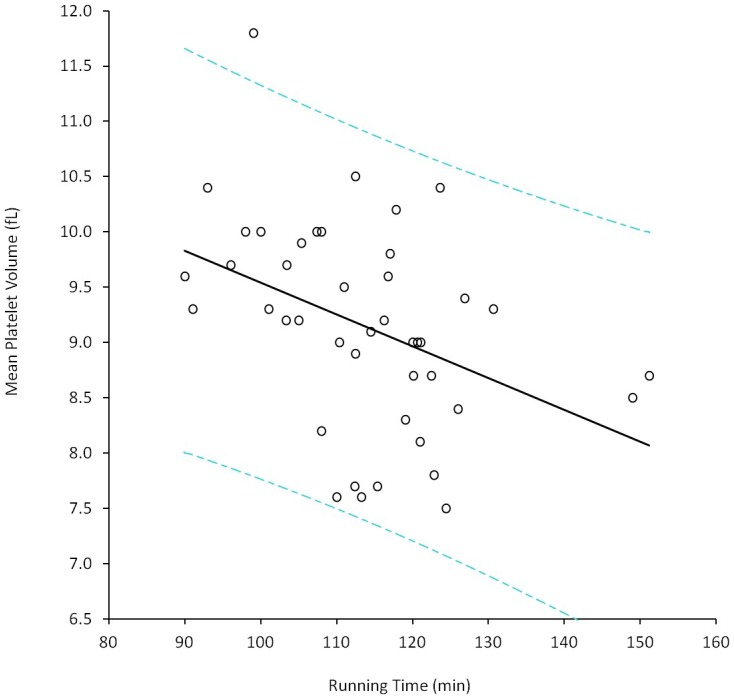
Correlation (and 95% prediction interval, 95% PI) between running performance and baseline value of mean platelet volume (MPV) in 43 amateur athletes completing a 21.1 km half-marathon run.

The 43 amateur runners completed the run in a median time of 113 min (IQR, 105–121 min). As predictable, the median running performance of the 28 male athletes (100 min and IQR 101–118 min) was significantly better than that of the 15 females athletes (120 min and IQR 113–123 min; p<0.001). The median body weight decreased by 2.2% after the run (from 73.1 to 71.5 kg; p<0.001). The median lactate value measured in capillary blood at the end of the run was 4.0 mmol/L (IQR, 3.0–4.9 mmol/L). The variation of the CBC parameters after the run is shown in table 1. The values of lymphocytes and eosinophils exhibited a significant decrease compared to pre-run values, whereas those of MCV, platelets, MPV, WBC, neutrophils and monocytes were found to be significantly increased after the run. In univariate analysis, significant predictors of finishing time were the pre-run values of hematocrit, hemoglobin, MCH, RDW, MPV, RetCHR, whereas the post-run values of MCH, RDW, MPV, monocytes and RetCHR were also associated with running performance (Table 2). The VO_2_max was the best overall predictor of running time (r = −0.601; p<0.001), whereas neither body mass index or blood lactate at the end of the half-marathon were significantly associated with running performance (Table 2). In multivariate analysis, in which running time was entered as dependent variable whereas age, sex, blood lactate, body mass index, VO_2_max, mean training regimen and the CBC parameters significantly associated with running performance in univariate analysis were entered as independent variables, only MPV values before and after the trial remained significantly associated with running time (Table 3). After adjustment for the platelet count, the MPV value before the run (p = 0.042), but not thereafter (p = 0.247), remained significantly associated with running performance (Fig. 1). Neither the platelet count (r = −0.210; p = 0.303) or the MPV (r = 0.039; p = 0.851) were significantly associated with VO_2_max in univariate analysis.

**Table 1:** Variation of the complete blood cell count after a 21.1 km half-marathon run in 43 amateur runners.

	Pre-run	Post-run	P
Hematocrit	0.45 (0.44–0.47)	0.45 (0.43–0.47)	0.420
Hemoglobin (g/L)	148 (140–155)	148 (138–155)	0.137
RBC (10^12^/L)	4.8 (4.6–5.0)	4.8 (4.5–5.1)	0.162
MCV (fL)	94 (91–96)	95 (92–97)	**0.004**
MCH (pg)	31 (30–32)	31 (30–32)	0.400
MCHC (g/dL)	32.7 (32.4–33.2)	32.5 (3.19–3.32)	0.068
RDW (%)	13.4 (13.1–13.5)	13.5 (13.1–13.6)	**0.001**
Platelets (10^9^/L)	260 (218–299)	321 (287–361)	**<0.001**
MPV (fL)	9.2 (8.6–9.8)	9.5 (8.9–10.1)	**<0.001**
WBC (10^9^/L)	5.6 (4.9–6.4)	12.4 (9.8–13.9)	**<0.001**
Neutrophils (10^9^/L)	3.1 (2.5–3.6)	9.3 (7.4–11.5)	**<0.001**
Lymphocytes (10^9^/L)	2.0 (1.7–2.3)	1.8 (1.5–2.2)	**0.037**
Monocytes (10^9^/L)	0.3 (0.2–0.4)	0.5 (0.4–0.6)	**<0.001**
Eosinophils (10^9^/L)	0.2 (0.1–0.2)	0.1 (0.0–0.01)	**<0.001**
Basophils (10^9^/L)	0.1 (0.1–0.1)	0.1 (0.0–0.1)	0.052
LUC (10^9^/L)	0.01 (0.1–0.1)	0.01 (0.1–0.1)	0.063
Reticulocytes (10^9^/L)	62 (54–74)	60 (52–73)	0.138
RetCHR (pg)	31 (31–32)	31 (31–32)	0.243

RBC, red blood cell; MCV, mean corpuscular volume; MCH, mean corpuscular hemoglobin (MCH); MCHC, mean corpuscular hemoglobin concentration; (MCHC); RDW, red blood cell distribution width; MPV, mean platelet volume (MPV); WBC, white blood cell; LUC, large unstained cells; RetCHR, reticulocyte hemoglobin concentration.

**Table 2:** Univariate correlation (r) analysis between running performance and parameters of the complete blood cell count in 43 amateur athletes who completed a 21.1 km half-marathon run.

	Pre-run value		Post-run value	
	r	p	r	p
Hematocrit	−0.329	**0.031**	−0.298	0.052
Hemoglobin	−0.388	**0.010**	−0.291	0.059
RBC	−0.074	0.635	−0.086	0.584
MCV	−0.234	0.131	−0.257	0.097
MCH	−0.306	**0.046**	−0.341	**0.025**
MCHC	−0.240	0.122	−0.199	0.200
RDW	0.316	**0.039**	0.336	**0.027**
Platelets	0.300	0.052	0.256	0.097
MPV	−0.450	**0.002**	−0.476	**0.001**
WBC	−0.208	0.181	0.248	0.109
Neutrophils	−0.142	0.365	0.262	0.090
Lymphocytes	−0.072	0.647	−0.028	0.861
Monocytes	−0.262	0.090	0.361	**0.017**
Eosinophils	−0.143	0.360	−0.258	0.095
Basophils	−0.096	0.538	−0.197	0.207
LUC	−0.039	0.805	0.185	0.234
Ret	0.290	0.059	0.208	0.181
RetCHR	−0.390	**0.001**	−0.379	**0.012**
Blood lactate	−	−	−0.069	0.663
Body mass index	0.092	0.555	-	-
VO2max (mL/min/Kg)	−0.601	**0.001**	-	-

RBC, red blood cell; MCV, mean corpuscular volume; MCH, mean corpuscular hemoglobin (MCH); MCHC, mean corpuscular hemoglobin concentration; (MCHC); RDW, red blood cell distribution width; MPV, mean platelet volume (MPV); WBC, white blood cell; LUC, large unstained cells; RetCHR, reticulocyte hemoglobin concentration; VO2max, maximal aerobic power.

**Table 3:** Multivariate correlation analysis between running performance and parameters of the complete blood cell count in 43 amateur athletes who completed a 21.1 km half-marathon run.

	Pre-run value	Post-run value
	p	p
Hematocrit	0.338	-
Hemoglobin	0.216	-
MCH	0.512	0.567
RDW	0.272	0.216
MPV	**0.042**	**0.026**
Monocytes	-	0.080
RetCHR	0.967	0.925

Results were also adjusted for age, sex, body mass index, post-run blood lactate, maximal aerobic power (VO_2_max) and training regimen.

MCH, mean corpuscular hemoglobin (MCH); RDW, red blood cell distribution width; MPV, mean platelet volume (MPV); RetCHR, reticulocyte hemoglobin concentration.

## Discussion

Due to the increasing popularity of recreational running as a form of leisure activity and health-promoting behavior, a large number of studies have been performed over the past decades to identify the most reliable predictors of running economy and performance. The large majority of these investigations focused on anthropometric variables, functional characteristics, as well as volume and intensity of training [13]. With the notable exception of hemoglobin and packed cell volume, little information is available on other hematological parameters that may predict middle distance running performance [14]. This investigation was hence specifically planned to establish whether some hematological parameters comprised within the CBC may be significantly associated with half-marathon running time.

The leukocytes variations recorded in this study are not new, since an increase of total leukocyte, neutrophil and monocyte counts along with a decrease of lymphocyte and eosinophils values have already been reported in a number of previous investigations, and are prevalently attributable to the well-documented release of catecholamines and cortisol during exercise [8], [15], [16].

The significant increase of both platelet count (median increase, 17%; IQR, 10–34%) and MPV (median increase, 6%; IQR, 1–9%) recorded immediately after the half-marathon run substantially exceeded the inter-individual biological variation of these parameters (platelet count, 9.1%; MPV, 4.3%) [17], and is also consistent with the well established evidence that aerobic physical activity is effective to enhance circulating activated platelets, as well as platelet-platelet and platelet-leukocyte aggregates [18]–[22]. More specifically, it has been recently demonstrated that the hyperactive platelets generated during exercise are rapidly cleared by the spleen, which is also a dynamic reservoir of younger and larger platelets (i.e., the human spleen retains one-third of total body platelets, with MPV approximately 20% greater than that of circulating platelets) [23]. The younger platelets are then released into the circulation, thus explaining the significant increase of platelet count and MPV observed after endurance exercise in this and other previous studies [18]–[22]. Another putative mechanism that may contribute to increase the MPV has been reported by Hilberg et al. [24], who observed that moderate exercise increased both platelet reactivity and platelet-leukocyte conjugate formation, which both contribute to increase the measured value of MPV. Regardless of the underlying mechanism(s), the significant increase of MPV recorded after exercise in this and other studies [18]–[22] has meaningful clinical implications, suggesting that the enhanced risk of cardiovascular events that is occasionally observed in athletes may be at least in part mediated by platelet hyper-reactivity [20]. Indeed, further studies are advisable to define whether an improvement of physical fitness is also accompanied with an increased MPV.

Interestingly, although the pre-run values of hematocrit, hemoglobin, MCH, RDW, MPV, RetCHR, along with the post-run values of MCH, RDW, MPV, monocytes and RetCHR were significantly associated with running time in univariate analysis, only the MPV values before and after the half-marathon remained significantly correlated with running performance in the fully-adjusted model. As predictable, both hemoglobin and hematocrit values were found to be positively correlated with running performance in univariate analysis, but the significance of these associations was lost in the fully adjusted model, especially when VO_2_max was entered as covariate. This is plausible, since VO_2_max and both hemoglobin and hematocrit clearly interplay in increasing sport performance, and VO_2_max is in fact enhanced by approximately 1% for each 3 g/L increase of hemoglobin [25].

As such, this is the first study demonstrating a direct correlation between platelet size and endurance performance to the best of our knowledge. It is noteworthy that the inverse association between pre-run MPV value and half-marathon running time remained significant after adjustment for a number of factors such as age, sex, blood lactate, body mass index, VO_2_max, mean training regimen and platelet count, thus confirming the existence of an effective interplay between platelet metabolism and aerobic performance. In univariate analysis, the correlation between running time and pre-run MPV value was the second highest overall, only preceded by that between running time and VO_2_max (Table 2). In agreement with a previous study [26], neither the platelet count or the MPV at baseline were significantly associated with VO_2_max, thus confirming that the influence of MPV on running performance may be virtually independent from the baseline cardiorespiratory fitness level.

An increased platelet volume is a well established surrogate marker of platelet activation, wherein large platelets are reportedly more active than small platelets [27]–[29]. The association of this evidence with our data would imply that platelet hyperactivity may be a significant determinant of performance in medium distance running. The use of platelets in sports medicine has risen sharply in recent times. The platelet-rich plasma (PRP), an autologous blood fraction rich in platelets and associated cytokines and growth factors, is mainly used for treatment of sports related injuries [30]–[32]. It was recently proven that injection of PRP may also exert some ergogenic effects. In particular, Wasterlain et al. studied the effect of PRP injection on variation of performance-enhancing systemic growth factors in 25 patients [33], and observed that the administration of PRP increased the concentration of insulin-like growth factor-1 (IGF-1), basic fibroblast growth factor (bFGF) and VEGF. Interestingly, Kasuya et al. also showed that a symptom-limited treadmill exercise test was effective to enhance the platelet release of nitric oxide (NO) [34], which would then contribute to raise exercise tolerance and performance [35].

Another mechanism by which platelets may contribute to enhance sport performance is the attenuation of neuropathic pain and/or fatigue during exercise [36]. Kennedy et al. studied platelet activation and function in 17 patients with chronic fatigue syndrome and 16 healthy controls [37], reporting that patients displayed lower platelet aggregability and reduced MPV. This would be consistent with the fact that smaller and less active platelets may somehow increase the fatigue threshold, thus conditioning exercise output. A series of studies also demonstrated that platelet gel or autologous platelet tissue graft are effective to lower pain after surgery and are associated with less pain medications and broader range of motion prior to discharge [38]–[40]. As specifically regards sports, the use of PRP was proven to be effective in reducing pain and promoting function improvement in tennis elbow [41] and other painful tendinopathies [42], as well as for accelerating muscle recovery after acute injury [43].

According to these evidences, it seems hence plausible that hyperactive platelets may exert some pleiotropic effects on endurance sport performance, by both releasing ergogenic mediators as well as by triggering an increase in performance-enhancing substances such as NO into the circulation. Further studies, involving also different running distances, sports and different categories of athletes are needed to confirm these findings and to elucidate the potential underlining mechanisms linking platelet volume and aerobic performance.
